# The impact of mal-angulated femoral rotational osteotomies on mechanical leg axis: a computer simulation model

**DOI:** 10.1186/s12891-020-3075-1

**Published:** 2020-01-23

**Authors:** Lukas Jud, Lazaros Vlachopoulos, Thomas V. Häller, Sandro F. Fucentese, Stefan Rahm, Patrick O. Zingg

**Affiliations:** Department of Orthopedics, Balgrist University Hospital, University of Zurich, Forchstrasse 340, 8008 Zürich, Switzerland

**Keywords:** Subtrochanteric osteotomy, Supracondylar osteotomy, Rotational osteotomy, Mechanical leg axis

## Abstract

**Background:**

Subtrochanteric or supracondylar femoral rotational osteotomies are established surgical treatments for femoral rotational deformities. Unintended change of the mechanical leg axis is an identified problem. Different attempts exist to plan a correct osteotomy plane, but implementation of the preoperative planning into the surgical situation can be challenging. Goal of this study was to identify the critical threshold of mal-angulation of the osteotomy plane and of femoral rotation that leads to a relevant deviation of the postoperative mechanical leg axis using a computer simulation approach.

**Methods:**

Three-dimensional (3D) surface models of the lower extremity of two patients (Model 1: 42° femoral antetorsion; Model 2: 6° femoral retrotorsion) were generated from computed tomography data. First, baseline subtrochanteric and supracondylar rotational osteotomies, perpendicular to the femoral mechanical axis were simulated. Afterwards, mal-angulated osteotomies in sagittal and frontal plane followed by different degrees of rotation were simulated and frontal mechanical axis was analyzed.

**Results:**

400 mal-angulated osteotomies have been simulated. Mal-angulation of ±30° with 30° rotation showed maximum deviation from preoperative mechanical axis in subtrochanteric osteotomies (4.0° ± 0.4°) and in supracondylar osteotomies (12.4° ± 0.8°). Minimal mal-angulation of 15° in sagittal plane in subtrochanteric osteotomies and mal-angulation of 10° in sagittal plane in supracondylar osteotomies altered the mechanical axis by > 2°. Mal-angulation in sagittal plane showed higher deviations of the mechanical axis (up to 12.4° ± 0.8°), than in frontal plane mal-angulation (up to 4.0° ± 1.9°).

**Conclusion:**

A femoral rotational osteotomy, perpendicular to the femoral mechanical axis, has no considerable influence on the mechanical leg axis. However, mal-angulation of femoral rotational osteotomies showed relevant changes of the mechanical leg axis. In supracondylar respectively subtrochanteric procedures, mal-angulation of only 10° in combination with already 15° of femoral rotation respectively mal-angulation of 15° in combination with 30° of femoral rotation, can lead to a relevant postoperative mechanical leg axis deviation of more than 2°, wherefore these patients probably would benefit from the use of navigation aids.

## Background

Femoral rotational deformities with excessive antetorsion or retrotorsion are frequently seen in patients with femoroacetabular impingement [1, 2], hip dysplasia [3, 4] or patellofemoral instability [5, 6]. Established treatment options are free-hand subtrochanteric or supracondylar femoral rotational osteotomies [7–9], bearing the risk of unintended changes in mechanical leg axis [10, 11]. Furthermore, a computer model study by Nelitz M et al. [12] showed a tendency to varus angulation in proximal- and a tendency to valgus angulation in distal femoral external-rotational osteotomies. In their study, the osteotomy plane was defined perpendicular to the femoral anatomical axis, probably the most common intraoperative landmark for orientation of the osteotomy plane. However, other authors propose to perform the osteotomy perpendicular to the femoral mechanical axis [13], possibly with less influence on the postoperative mechanical leg axis. There are different other attempts for preoperative planning of the correct osteotomy plane in femoral rotational osteotomies [14–16]. Nonetheless, implementation of the preoperative planning into the surgical situation can be challenging, wherefore some deviation from the planning is likely in most cases. A possible remedy could be the use of patient specific instruments (PSI) [15, 17]. However, PSI are not yet routinely used in such surgical procedures and they are not always available. Moreover, the additional costs of PSI need to be considered. Probably their use should, however, be considered in risk-prone patients, such as cases with the need of higher degrees of femoral rotation.

So far no study exists that investigates the change of the mechanical leg axis in case of a femoral rotational osteotomy perpendicular to the femoral mechanical axis, and that assess the effect of an unintentionally mal-angulated osteotomy plane. Therefore, three-dimensional (3D) patient models with excessive femoral antetorsion and retrotorsion were used to simulate subtrochanteric and supracondylar rotational osteotomies with different angulated osteotomy planes and different degrees of rotation. As an intended correction of the mechanical leg axis in high tibial osteotomy shows accuracy of about 2° [18], a postoperative mechanical leg axis deviation of more than 2° was defined as a relevant mechanical axis deviation. Using this computer simulation approach, it was the goal of this study to investigate a femoral rotational osteotomy perpendicular to the femoral mechanical axis and to identify the critical threshold of mal-angulation and femoral rotation that leads to a relevant deviation in postoperative mechanical leg axis of more than 2°, respectively to identify surgical procedures that are more risk-prone for relevant postoperative mechanical leg axis deviation and therefore would benefit from the use of navigation aids (e.g. PSI).

## Methods

**Fig. 1:**
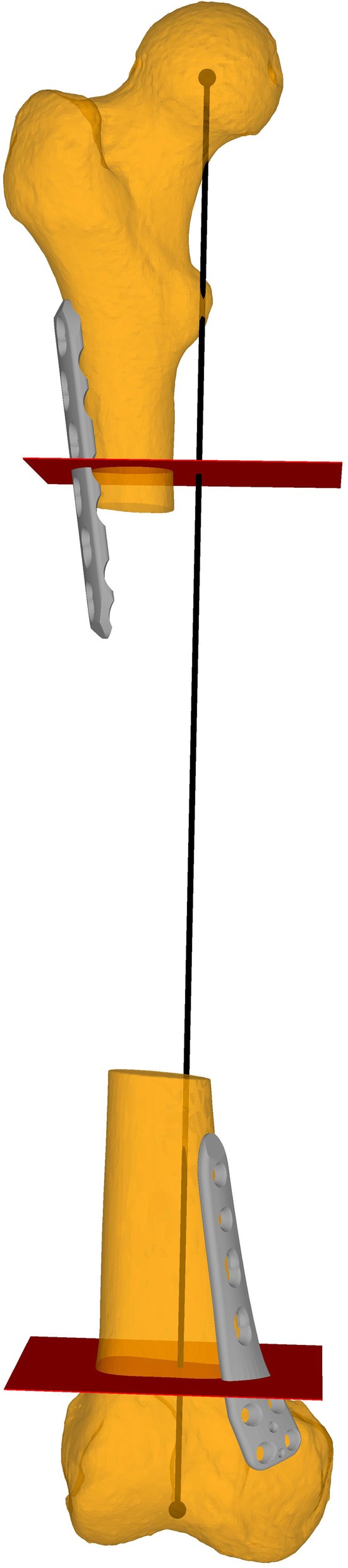
Baseline osteotomy planes (i.e. perpendicular to the femoral mechanical axis). In red the subtrochanteric and the supracondylar osteotomy. In grey the 6 holes 4.5 mm Broad LCP Plate and the TomoFix Medial Distal Femur Plate. In black the mechanical femoral axis

**Fig. 2:**
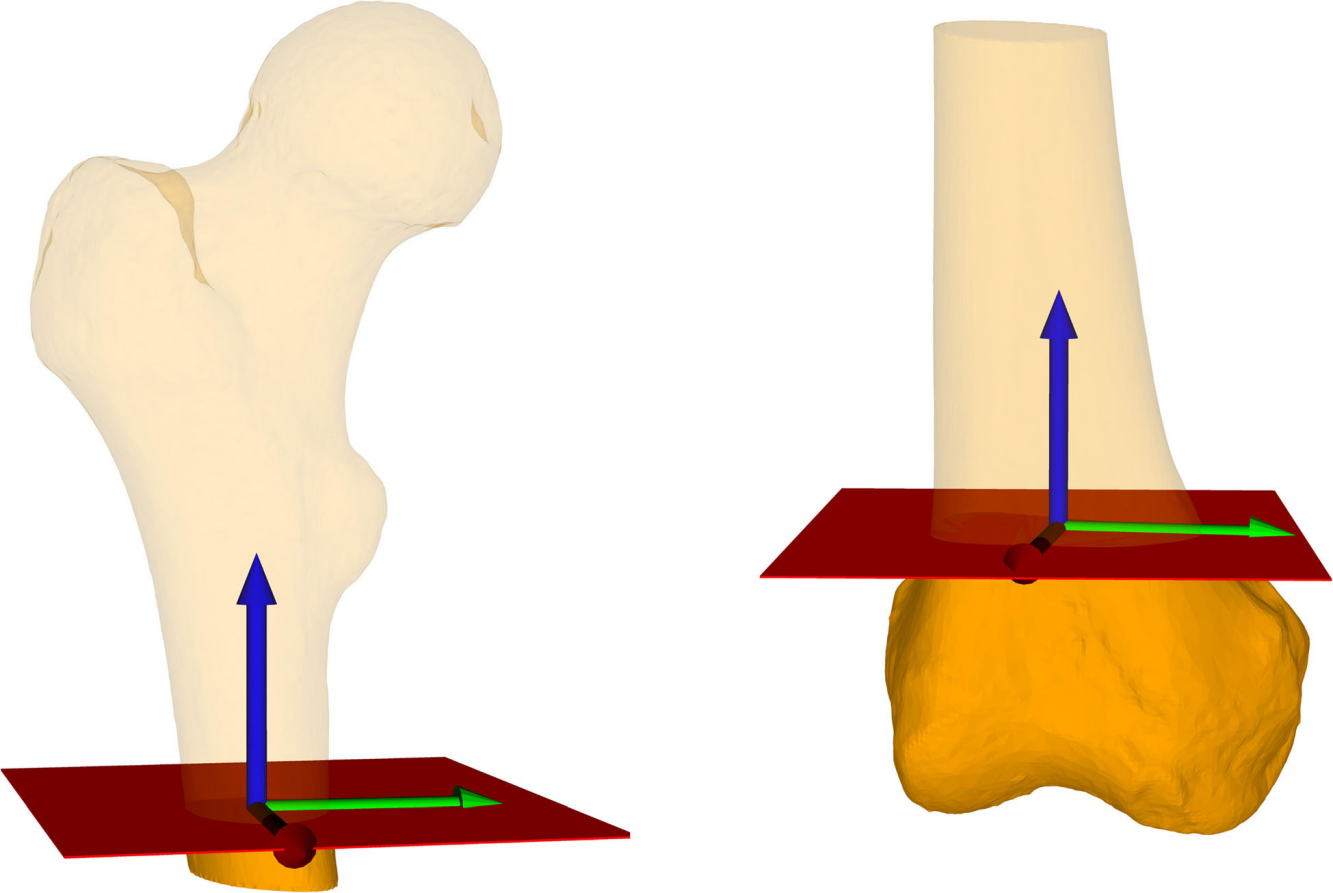
Coordinate systems. In red the baseline osteotomy planes. A coordinate system in the middle of the femoral shaft on the level of the osteotomy-plane was defined. According to the y-axis (blue) internal- respectively external rotation of the distal fragment was performed. On the left side the z-axis (green) is a projection of the femoral neck axis on the osteotomy plane. On the right side the z-axis (green) is a projection of a tangent to the posterior femoral condyles on the osteotomy plane. The x-axis (red) in both figures is the cross product of the y- and z-axis

**Fig. 3:**
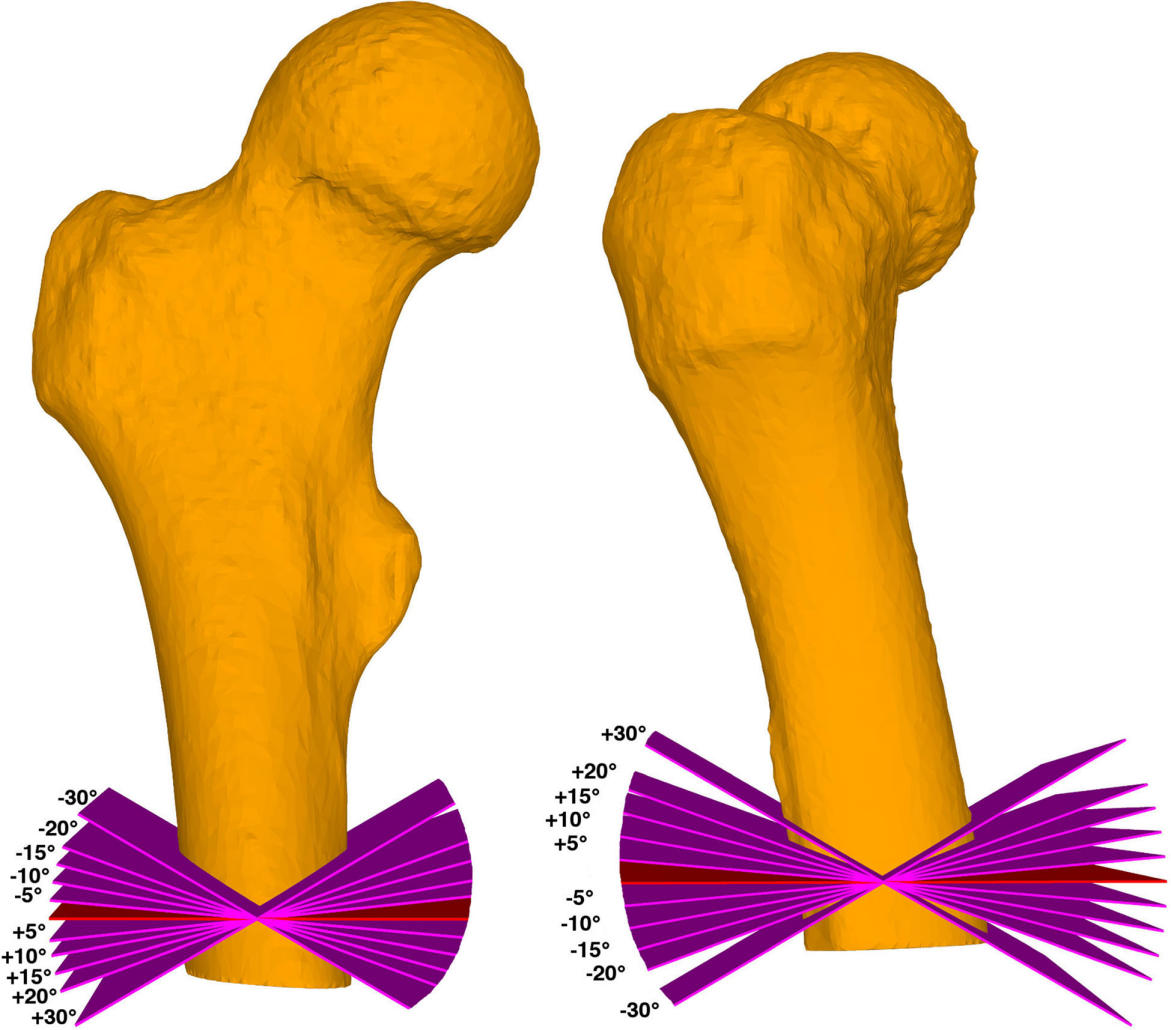
Mal-angulated osteotomies. Mal-angulated osteotomy planes in subtrochanteric procedures: on the left side in frontal plane, on the right side in the sagittal plane. In red the baseline osteotomy planes, in purple the mal-angulated osteotomy planes

3D surface models of the lower extremity of the right side of a patient with femoral antetorsion (42 degrees of antetorsion, Model 1) and of a patient with femoral retrotorsion (6 degrees of retrotorsion, Model 2) were generated from computed tomography (CT) data. Besides the rotational deformity, both used patient models had a normal femoral anatomy with a femoral antecurvatum angle of 8° in Model 1 and 14° in Model 2 and a mechanical lateral distal femoral angle (mLDFA) of 85° respectively 86°. The bone models were imported into the in-house developed surgical planning software CASPA (Balgrist CARD AG, Zurich, Switzerland). Measuring the antero-posterior (AP)-projected 3D mechanical leg axis, using a measurement method similar to the one described by Fürnstahl et al. [19], showed 2.4° valgus for Model 1 and 5.1° valgus for Model 2. A line segment connecting the center of the femoral head and the center of the intercondylar notch represented the femoral mechanical axis. The baseline subtrochanteric and supracondylar osteotomy plane was defined to be perpendicular to the mechanical femoral axis [13]. The level of the subtrochanteric osteotomy was set 45 mm below the lesser trochanter in Model 1 and 40 mm below the lesser trochanter in Model 2, in a way that a 6 holes 4.5 mm Broad LCP Plate (Depuy-Synthes Oberdorf, Switzerland) could be properly placed (Fig. 1). Likewise the level of the supracondylar osteotomy was set 60 mm above the femoral condyles in Model 1 and 65 mm above the femoral condyles in Model 2, in a way that a TomoFix Medial Distal Femur Plate (Depuy-Synthes Oberdorf, Switzerland) could be properly placed (Fig. 1). A standardized coordinate system was defined to place the mal-angulated osteotomy planes and to analyze their effect on the mechanical leg axis. The geometrical center of a narrow slice at the level of the osteotomy was selected as the center of the coordinate system (Fig. 2). Orientation of the axes were defined according to the International Society of Biomechanics (ISB) recommendation on definitions of joint coordinate systems [20] wherefore the y-axis was defined as the direction of the mechanical femoral axis. For the subtrochanteric osteotomies, the z-axis was defined as the projection of the femoral neck axis on the osteotomy plane, pointing medially. Reason therefore was the lateral surgical approach to the subtrochanteric region. For the supracondylar osteotomies, the z-axis was defined as the projection of a tangent to the posterior femoral condyles on the osteotomy plane, pointing medially. Reason therefore was beside the surgical approach, the intraoperative reference-orientation on the posterior femoral condyles. The x-axis was the cross product of the y- and z-axis pointing anteriorly. Subtrochanteric and supracondylar osteotomies were consecutively performed in both models with the baseline osteotomy planes. Following mal-angulated osteotomy planes were defined with angulation in frontal plane (x-axis) and sagittal plane (z-axis) in steps of 5°, 10°, 15°, 20°, and 30° in clockwise and counter-clockwise directions in relation to the defined reference-coordinate system of the subtrochanteric respectively the supracondylar osteotomy plane (Fig. 3). In Model 1, for the baseline and all mal-angulated osteotomy planes, external rotation of the distal femoral fragment on the osteotomy plane was performed in steps of 5°, 10°, 15°, 20°, and 30°. For each position the 3D mechanical leg axis was calculated, as previously described. Likewise in Model 2, for the baseline and all mal-angulated osteotomy planes, internal rotation of the distal femoral fragment on the osteotomy plane was performed in steps of 5°, 10°, 15°, 20°, and 30° with calculation of each 3D mechanical leg axis.

For interpretation of the data, positive values of the mechanical leg axis have been handled as valgus, and negative values as varus.

The local ethical committee approved this study (Zurich Cantonal Ethics Commission, Req-2019-00133), and the patients gave their informed consent for their participation and the publication of the data.

### Statistical analysis

Descriptive analysis was performed. Statistical calculation of mean values and standard deviation was carried out with the software R (version 1.1.463; R foundation, Vienna, Austria).

## Results

For each model subtrochanteric and supracondylar osteotomies with the baseline osteotomy planes were simulated with subsequent five different degrees of rotation, resulting in 20 rotational osteotomies. Overall 80 mal-angulated osteotomy planes have been generated, and likewise five different degrees of rotations were performed, resulting in 400 simulations with mal-angulated rotational osteotomies.

With the baseline osteotomy planes, the different simulated rotations altered the mechanical axis by a mean of 0.1° ± 0.2°. In Model 1 and Model 2, postoperative mean mechanical leg axis was 2.4° ± 0.1° and 4.9° ± 0.3° compared to the preoperative axis of 2.4° and 5.1°, respectively.

An overview of mean deviation from preoperative mechanical leg axis for the particular angulation failure and for the different degrees of rotation in subtrochanteric osteotomies is given in Table 1 and for supracondylar osteotomies in Table 2.

**Table 1:** Deviations from preoperative mechanical leg axis in subtrochanteric osteotomies

Angulation Error of the Osteotomy Plane	Plane	Error in the Mechanical Leg Axis Alignment per Rotation				
		5°	10°	15°	20°	30°
+/− 5°	Sagittal	0.2° ± 0.2°	0.3° ± 0.3°	0.4° ± 0.3°	0.5° ± 0.4°	0.7° ± 0.5°
	Frontal	0.1° ± 0.1°	0.2° ± 0.1°	0.2° ± 0.1°	0.3° ± 0.2°	0.4° ± 0.2°
+/− 10°	Sagittal	0.2° ± 0.1°	0.5° ± 0.2°	0.7° ± 0.3°	0.9° ± 0.3°	1.4° ± 0.4°
	Frontal	0.1° ± 0.0°	0.2° ± 0.1°	0.3° ± 0.1°	0.3° ± 0.1°	0.4° ± 0.3°
+/− 15°	Sagittal	0.3° ± 0.1°	0.7° ± 0.2°	1.0° ± 0.3°	1.4° ± 0.3°	**2.1° ± 0.4°**
	Frontal	0.1° ± 0.0°	0.2° ± 0.1°	0.3° ± 0.1°	0.4° ± 0.1°	0.5° ± 0.3°
+/− 20°	Sagittal	0.5° ± 0.1°	0.9° ± 0.2°	1.4° ± 0.3°	**1.8° ± 0.3°**	**2.8° ± 0.4°**
	Frontal	0.2° ± 0.0°	0.3° ± 0.1°	0.4° ± 0.0°	0.5° ± 0.0°	0.5° ± 0.2°
+/− 30°	Sagittal	0.7° ± 0.1°	1.3° ± 0.2°	**2.0° ± 0.2°**	**2.7° ± 0.3°**	**4.0° ± 0.4°**
	Frontal	0.2° ± 0.1°	0.3° ± 0.1°	0.5° ± 0.1°	0.6° ± 0.1°	0.6° ± 0.2°

Errors in mechanical leg axis greater than 2° (mean value plus standard deviation) have been marked bold

**Table 2:** Deviations from preoperative mechanical leg axis in supracondylar osteotomies

Angulation Error of the Osteotomy Plane	Plane	Error in the Mechanical Leg Axis Alignment per Rotation				
		5°	10°	15°	20°	30°
+/− 5°	Sagittal	0.4° ± 0.0°	0.8° ± 0.0°	1.1° ± 0.1°	1.5° ± 0.1°	2.1° ± 0.2°
	Frontal	0.1° ± 0.1°	0.1° ± 0.1°	0.2° ± 0.2°	0.4° ± 0.2°	0.8° ± 0.3°
+/− 10°	Sagittal	0.8° ± 0.0°	1.5° ± 0.1°	**2.2° ± 0.1°**	**2.9° ± 0.2°**	**4.2° ± 0.3°**
	Frontal	0.1° ± 0.1°	0.3° ± 0.2°	0.5° ± 0.3°	0.8° ± 0.4°	**1.5° ± 0.6°**
+/− 15°	Sagittal	1.1° ± 0.0°	**2.2° ± 0.1°**	**3.3° ± 0.2°**	**4.4° ± 0.2°**	**6.3° ± 0.4°**
	Frontal	0.2° ± 0.1°	0.4° ± 0.3°	0.7° ± 0.5°	1.1° ± 0.7°	**2.2° ± 1.0°**
+/− 20°	Sagittal	1.5° ± 0.1°	**2.9° ± 0.1°**	**4.4° ± 0.2°**	**5.8° ± 0.3°**	**8.4° ± 0.6°**
	Frontal	0.2° ± 0.2°	0.5° ± 0.5°	0.9° ± 0.7°	**1.4° ± 0.9°**	**2.9° ± 1.3°**
+/− 30°	Sagittal	**2.2° ± 0.1°**	**4.3° ± 0.2°**	**6.4° ± 0.3°**	**8.5° ± 0.4°**	**12.4° ± 0.8°**
	Frontal	0.3° ± 0.3°	0.7° ± 0.7°	**1.3° ± 1.0°**	**2.0° ± 1.3°**	**4.0° ± 1.9°**

Errors in mechanical leg axis greater than 2° (mean value plus standard deviation) have been marked bold

In general, higher degree of deviation from preoperative mechanical leg axis could be observed with higher degrees of rotation and with higher degrees of mal-angulation.

**Fig. 4:**
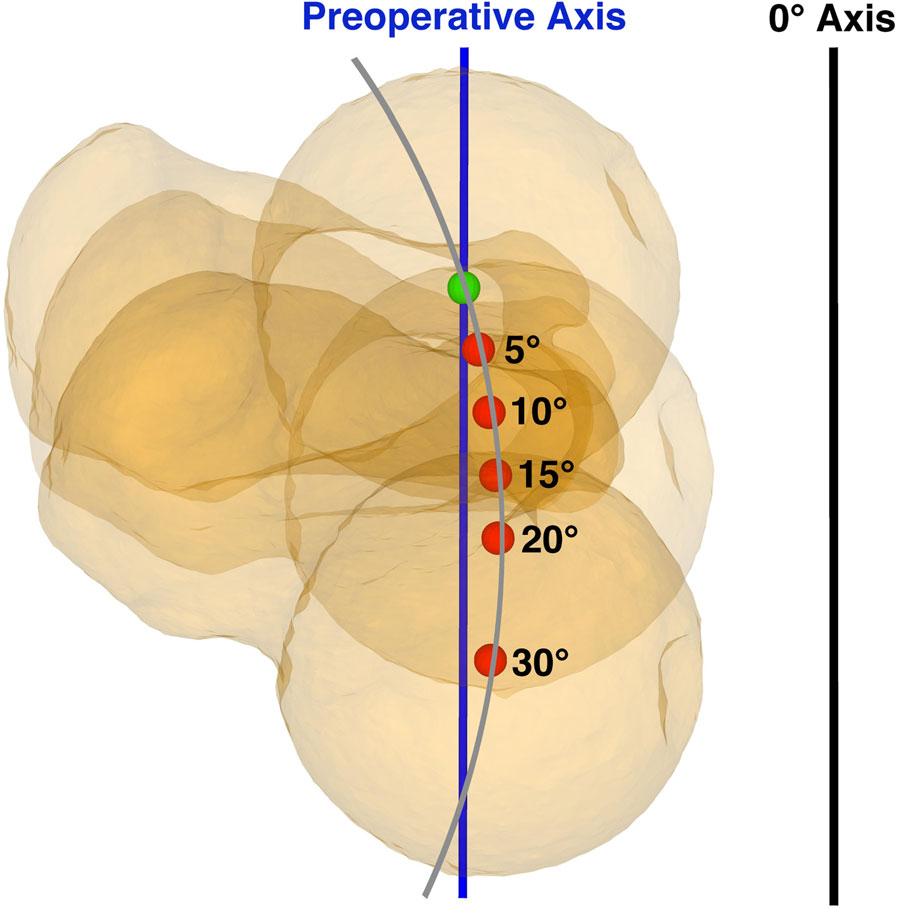
Decrease of postoperative axis deviation. Illustration of the decrease of postoperative deviation of mechanical leg axis in case of mal-angulation in counter-clockwise direction in frontal plane in subtrochanteric rotational osteotomy. With rotation over 20° a decrease of postoperative deviation of AP-projected mechanical leg axis could be observed. In green the preoperative hip-center, in red the hip-centers for each rotation, in black the AP-projected 0° varus/valgus axis, in grey the circle of relative rotation of the hip-center during rotational osteotomy

However, an exception could be observed in mal-angulation in subtrochanteric osteotomies with mal-angulation in counter-clockwise direction in the frontal plane. In these cases an increasing deviation of the postoperative axis could be observed with rotation up to 20°. With 30° of rotation a decrease of deviation could be observed, caused by the relative circular movement of the hip-center during rotation. The peak of the circle (i.e. maximum distance between initial hip-center and circle of movement of the hip-center during rotation) was reached with 20° of rotation, and with higher degrees of rotation the hip-center moved back to the initial AP-projected preoperative hip-center, whereas a decrease of changes of mechanical leg axis could be observed. This effect is illustrated in Fig. 4. All other simulated osteotomies resulted in a relative circular movement of the hip-center, with rotation up to 30°, away from the preoperative hip-center and therefore with an increase of deviation of postoperative mechanical leg axis.

## Discussion

The most important finding of the present study was that a femoral rotational osteotomy, perpendicular to the femoral mechanical axis, has no considerable influence on the mechanical leg axis but a deviation from this baseline position can result in a relevant deviation of the postoperative mechanical leg axis in dependence of the degree of femoral rotation. In supracondylar osteotomy, a mal-angulation of already 10° and rotation of 15° lead to a relevant mechanical leg axis deviation of 2.2° ± 0.1°. In subtrochanteric osteotomy, relevant axis deviation could be observed from mal-angulation of 15° and rotation of 30°, with mean values of 2.1° ± 0.4°. Overall with increasing angulation failure, as well as with increasing rotations, more influence on mechanical leg axis could be observed with maximum mean deviations up to 12.4° ± 0.8° (supracondylar osteotomies with mal-angulation of ±30° in sagittal plane and rotation of 30°).

**Fig. 5:**
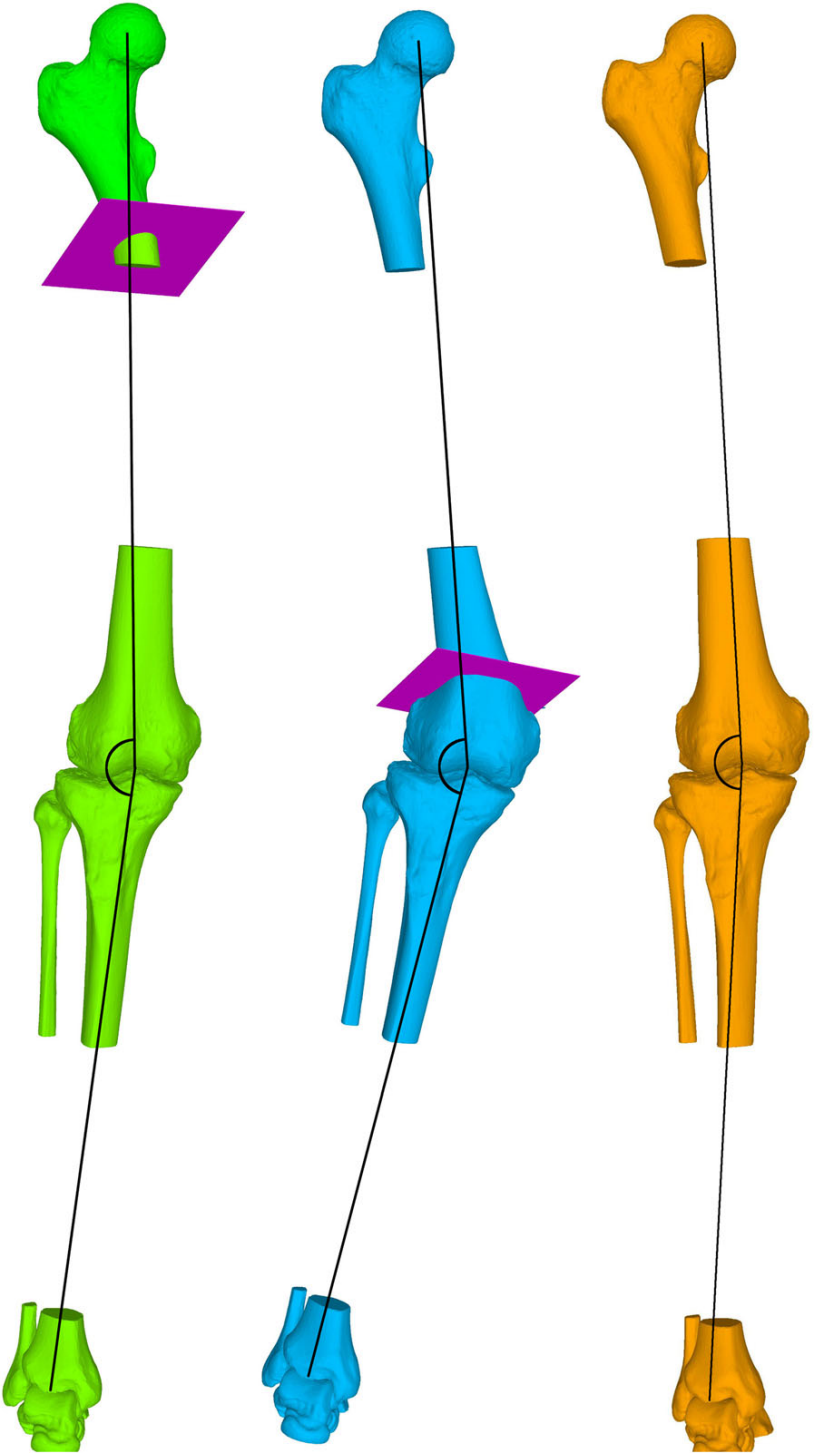
Illustration of the marked difference in postoperative axis deviation between subtrochanteric and supracondylar osteotomies. In green subtrochanteric osteotomy with mal-angulation of 30° in sagittal plane and rotation of 30° resulting in a postoperative mechanical leg axis of 8.6° valgus (preoperative axis 5.1° valgus). In blue supracondylar osteotomy with mal-angulation of 30° in sagittal plane and rotation of 30° resulting in a postoperative mechanical leg axis of 18.2° valgus (preoperative axis 5.1° valgus). In orange visualized the preoperative situation. For visualization purposes the mechanical leg axis has been marked schematically in black

In general, mal-angulation in sagittal plane showed to be more vulnerable for relevant changes of mechanical leg axis (up to mean deviations of 12.4° ± 0.8° in supracondylar osteotomies) than mal-angulation in frontal plane (up to mean deviations of 4.0° ± 1.9° in supracondylar osteotomies). The same applies for mal-angulation in supracondylar osteotomies with higher degrees of deviations from preoperative mechanical leg axis (up to mean deviations of 12.4° ± 0.8° in sagittal plane mal-angulation) than in subtrochanteric osteotomies (up to mean deviations of 4.0° ± 0.4° in sagittal plane mal-angulation) (Fig. 5).

The marked difference in postoperative deviation of mechanical leg axis between subtrochanteric and supracondylar osteotomies can be explained according to Nelitz M. et al. [12] who described a tendency to varus angulation in case of proximal femoral rotational osteotomies and the tendency to valgus angulation in distal femoral rotational osteotomies. Proximal femoral rotational osteotomy more affects the AP-projected relative femoral neck length wherefore the generated deviation through the mal-angulated osteotomy gets partially compensated. In distal femoral rotational osteotomy the center of rotation is closer to the mechanical femoral axis wherefore this effect is less pronounced and the deviation of postoperative mechanical leg axis by mal-angulated osteotomy becomes more remarkable.

Avoidance of unintended alteration of the mechanical leg axis is crucial, as it is known that varus- or valgus-malalignment may result in either overload of the medial or lateral knee compartment, provoke patellar maltracking or may aggravate symptoms in knee arthritis [21, 22]. However, with conventional surgical technique, intraoperative estimation of a perpendicular osteotomy plane to an imagined axis (i.e. the mechanical femoral axis) may be challenging. The limited surgical exposure additionally aggravates this challenge. Therefore, with the relevant alteration of the mechanical axis with mal-angulation of the osteotomies demonstrated in this study, an accurate preoperative planning and probably the use of intraoperative navigations aids should be considered for such surgical procedures, to properly implement the preoperative planning into the intraoperative situation, and to prevent deviation of postoperative mechanical leg axis. One possible solution could be a preoperative 3D planning and the use of PSI, already described for femoral rotational osteotomies by Fiz et al. [17]. In particular these considerations should be taken in account in cases with higher degrees of rotations, as well as in cases with supracondylar procedures.

This study has several limitations. First, there was a simulation of isolated angulation failures in the sagittal and frontal plane only. A combination of sagittal and frontal plane mal-angulation probably increases the postoperative mechanical leg axis deviation or possibly compensates each other. Second, only the AP-projected mechanical leg axis was investigated. As it is known that femoral antecurvatum angle is affected by femoral rotational osteotomies [12], it has to be assumed that it is also influenced by mal-angulated femoral osteotomies. Goal of the present study was to investigate the change of the AP-projected mechanical leg axis, as it is the most utilized parameter in daily practice, assessing the leg axis of a patient. Investigating changes in femoral antecurvatum angle in case of mal-angulated femoral rotational osteotomies is the aim of future studies. Third limitation of this study is the use of only two patient models, one with femoral antetorsion of 42°, and one with femoral retrotorsion of 6°, presumably covering the range of rotational deformities in daily practice. In case of higher degrees of deformity and with higher degrees of rotation, possibly more deviation of the postoperative mechanical leg axis can be expected. Probably the same applies to more pronounced preoperative mechanical leg axis deformities. Fourth, it has to be mentioned that for example a rotation of 30° in case of 6° femoral retrotorsion would result in an overcorrection. These hypothetical corrections were performed for the sake of completeness of the spectrum of rotations in the utilized limited model in this study.

## Conclusion

A femoral rotational osteotomy, perpendicular to the femoral mechanical axis, has no considerable influence on the mechanical leg axis. However, mal-angulation of femoral rotational osteotomies showed relevant changes of the mechanical leg axis. In supracondylar respectively subtrochanteric procedures, mal-angulation of only 10° in combination with already 15° of femoral rotation respectively mal-angulation of 15° in combination with 30° of femoral rotation, can lead to a relevant postoperative mechanical leg axis deviation of more than 2°, wherefore these patients probably would benefit from the use of navigation aids.

## Data Availability

Anonymized source data can be obtained from the corresponding author on reasonable request.

## Abbreviations

3D: Three-dimensional
AP: Antero-posterior
CT: Computed tomography
mLDFA: mechanical lateral distal femoral angle
PSI: Patient specific instruments
